# *In vivo* production of non-glycosylated recombinant proteins in *Nicotiana benthamiana* plants by co-expression with Endo-β-N-acetylglucosaminidase H (Endo H) of *Streptomyces plicatus*

**DOI:** 10.1371/journal.pone.0183589

**Published:** 2017-08-21

**Authors:** Tarlan Mamedov, Kader Cicek, Burcu Gulec, Rifat Ungor, Gulnara Hasanova

**Affiliations:** Akdeniz University, Department of Agricultural Biotechnology, Antalya, Turkey; University of Canterbury, NEW ZEALAND

## Abstract

A plant transient expression system, with eukaryotic post-translational modification machinery, offers superior efficiency, scalability, safety, and lower cost over other expression systems. However, due to aberrant N-glycosylation, this expression system may not be a suitable expression platform for proteins not carrying N-linked glycans in the native hosts. Therefore, it is crucial to develop a strategy to produce target proteins in a non-glycosylated form while preserving their native sequence, conformation and biological activity. Previously, we developed a strategy for enzymatic deglycosylation of proteins *in planta* by co-expressing bacterial peptide-N-glycosidase F (PNGase F). Though PNGase F removes oligosaccharides from glycosylated proteins, in so doing it causes an amino acid change due to the deamidation of asparagine to aspartate in the N-X-S/T site. Endo-β-*N*-acetylglucosaminidase (EC3.2.1.96, Endo H), another deglycosylating enzyme, catalyzes cleavage between two N-Acetyl-D-glucosamine residues of the chitobiose core of *N*-linked glycans, leaving a single N-Acetyl-D-glucosamine residue without the concomitant deamidation of asparagine. In this study, a method for *in vivo* deglycosylation of recombinant proteins in plants by transient co-expression with bacterial Endo H is described for the first time. Endo H was fully active *in vivo*. and successfully cleaved N-linked glycans from glycoproteins were tested. In addition, unlike the glycosylated form, *in vivo* Endo H deglycosylated Pfs48/45 was recognized by conformational specific Pfs48/45 monoclonal antibody, in a manner similar to its PNGase F deglycosylated counterpart. Furthermore, the deglycosylated PA83 molecule produced by Endo H showed better stability than a PNGase F deglycosylated counterpart. Thus, an Endo H *in vivo* deglycosylation approach provides another opportunity to develop vaccine antigens, therapeutic proteins, antibodies, and industrial enzymes.

## Introduction

Plant based transient expression system is a promising technology for the production of various recombinant proteins including vaccine antigens, therapeutic proteins, antibodies and industrial enzymes. These systems offer superior benefits over other expression systems, which include: quick production timeline, low cost input, highly scalable, high production capacity and they do not harbor mammalian pathogens. In addition, because plants have eukaryotic post-translational modifications [1], including N-linked glycosylation, the technique may be particularly useful for the expression of glycosylated proteins including mammalian proteins, in which N-glycosylaton is critical for their functional activity. However, the ability of plants to glycosylate proteins can be a significant limitation for the production of proteins not carrying N-linked glycans in the native hosts such as Pfs48/45 protein of *Plasmodium falciparum*, the A chain of human factor XIII, or the protective antigen (PA) of *Bacillus anthracis*.These proteins are not glycoproteins in the native hosts, however, they contain potential N-linked glycosylation sites that can be aberrantly glycosylated during production in any eukaryotic expression system. Therefore, we sought to develop a robust strategy to produce recombinant proteins in a non-glycosylated form, while preserving their native amino acid sequence, conformation. and biological activity. Recently, we developed a strategy of enzymatic deglycosylation of proteins *in planta* by co-expressing bacterial PNGase F (Peptide: N-glycosidase F) [2,3]. Using this strategy, Pfs48/45 protein was produced in *Nicotiana benthamiana* in a non-N-glycosylated form and conformation-specific mAbs, produced against different epitops of native Pfs48/45 protein, recognized the deglycosylated form of Pfs48/45 2- to 6-fold better than they recognized the glycosylated form [2]. Furthermore, plant produced deglycosylated PA83 was found to be more stable and elicited significantly higher levels of toxin-neutralizing antibody titers in immunized mice compared to its glycosylated plant produced PA83 counterpart [4]. In addition, the lethal toxin neutralizing activity and immunogenicity of plant produced *in vivo* deglycosylated PA83 was much higher than those of *in vitro* deglycosylated PA83, or mutant forms, indicating potential differences in protein folding of *in vivo*, *in vitro* or mutant non-glycosylated forms [4].

**Fig 1:**
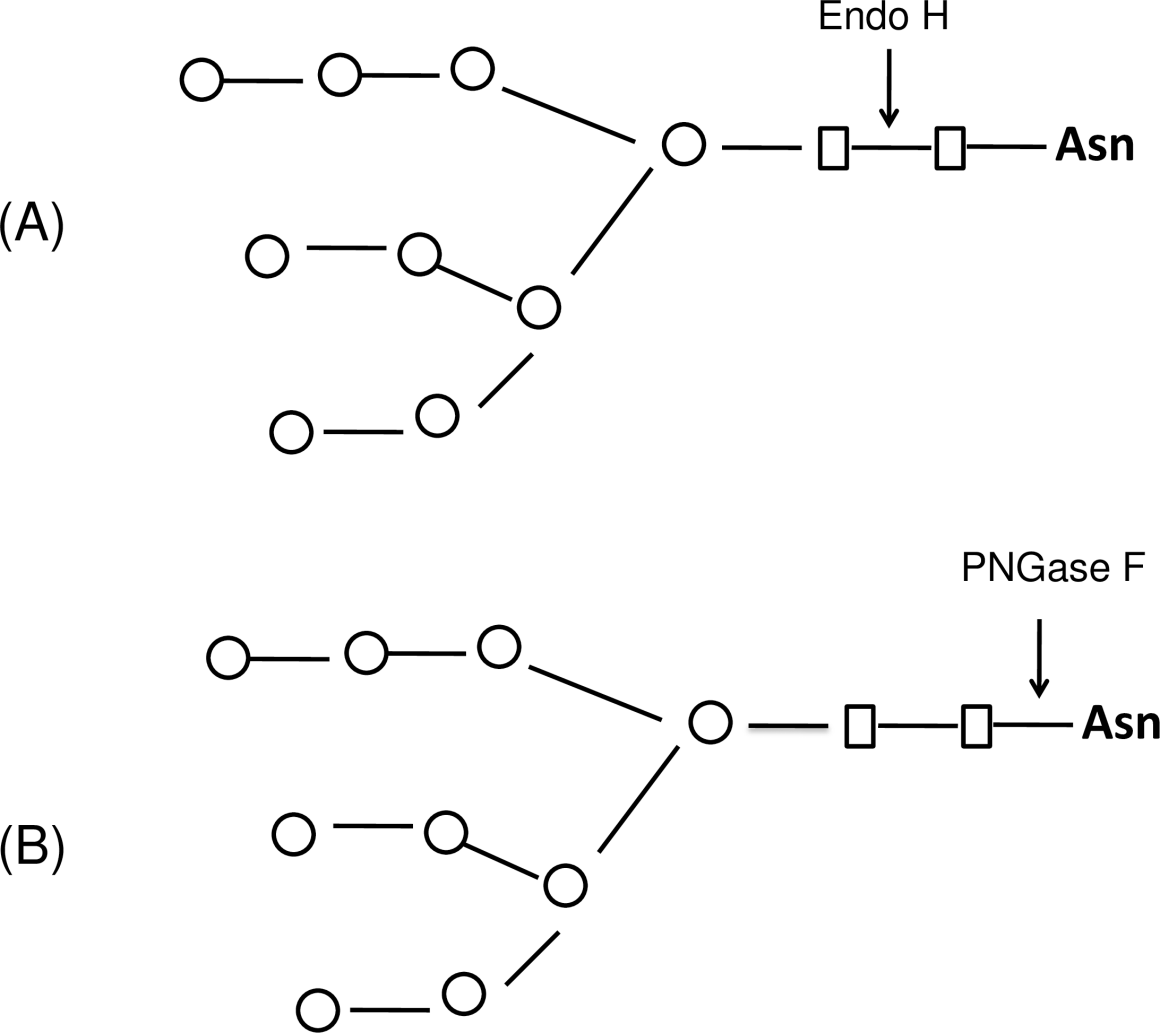
Schematic representation of Endo H or PNGase F cleavages. (A) Endo H cleaves between the two GlcNAc residues in the diacetylchitobiose core of the oligosaccharide, generating a truncated sugar molecule with one GlcNAc remaining on the asparagines (Asn). (B) Peptide -*N*-Glycosidase F (PNGase F), is an amidase that cleaves the bond between GlcNAc and asparagine residues of high mannose, hybrid, and complex oligosaccharides from *N*-linked glycoproteins;

Though deglycosylated proteins, produced by co-expression with PNGase F showed superior functional properties over their glycosylated counterparts, PNGase F (*in vivo or in vitro* deglycosylation) does cause an amino acid change in the deglycosylated protein at the glycosylation site (N-X-S/T) due to the deamidation of asparagine to aspartate [2,5]. Another deglycosylating enzyme, Endo-β-*N*-acetylglucosaminidase H (EC 3.2.1.96, Endo H) catalyzes the cleavage of the β-1, 4-glycosidic bond between two N-Acetyl-D-glucosamine (GlcNAc) residues with great efficiency in the diacetylchitobiose core of *N*-linked glycans, leaving a single GlcNAc residue attached to asparagines [6,7] with no concomitant deamidation [5,8] (Fig 1). This enzyme cleaves both high mannose and hybrid N-linked glycans, however, does not cleave the complex N-linked glycans from asparagine-linked glycoproteins [9]. Endo H, isolated from the culture *S*. *plicatus* as monomeric polypeptide, has a molecular weight of about 27 kDa [10]. A number of studies demonstrated that Endo H can be widely used to study the glycoprotein functions and their structures and to estimate the number of N-linked glycans of glycoproteins [11–13]. Recombinant Endo H from *S*. *plicatus* was recently expressed in *Pichia pastoris* and its activity was demonstrated *in vitro*, through both co-fermentation and post-fermentation treatments [14]. However, N-deglycosylation of proteins *in vivo* by Endo H enzyme has not been achieved yet. In this study, we developed a method for producing deglycosylated proteins in *N*. *benthamiana* plants *in vivo*, by transiently expressing bacterial Endo H with a target protein of interest. Our results show that Endo H is fully active *in vivo* and *in vitro* and successfully cleaved N-linked glycans from malaria vaccine antigens Pfs48/45 or Pfs48/45-10C (a truncated form of Pfs48/45) [15] and PA of *B*. *anthracis*. Furthermore, we demonstrate that, unlike the glycosylated form, *in vivo* Endo H deglycosylated Pfs48/45 was recognized by conformational specific Pfs48/45 monoclonal antibody, a known transmission blocking (TB) antibody, in a manner similar to its PNGase F deglycosylated counterpart; however, the deglycosylated PA83 molecule produced by Endo H showed better stability than the PNGase F deglycosylated counterpart.

## Materials and methods

### Cloning, expression and purification of recombinant Endo H produced in *N*. *benthamiana* plants, and evaluation of its deglycosylating activity *in vitro*

The Endo H gene (GenBank accession AAA26738.1) was optimized for expression in *N*. *benthamiana* plants and synthesized by GENEART AG (Thermo Fisher Scientific). To express Endo H in *N*. *benthamiana* plants, the signal peptide (amino acids 1–42) was replaced with the *Nicotiana tabacum* PR-1a signal peptide (MGFVLFSQLPSFLLVSTLLLFLVISHSCRA). In addition, the ER retention signal (KDEL) and the FLAG epitope (DYKDDDDK) coding sequences were added to the C-terminus. The PNGase F gene was also co-expressed with target proteins for comparison of deglycosylation of target proteins by Endo H. The sequence of PNGase F (GenBank accession number J05411) was optimized for expression in *N*. *benthamiana* plants and synthesized at Integrated DNA Technologies (IDT) with the FLAG epitope and a C-terminal ER retention peptide, KDEL. Non-tagged versions of the Endo H and PNGase F genes, with KDEL but lacking the FLAG epitope, were also constructed to avoid co-elution of plant produced recombinant Endo H or PNGase F with FLAG- tagged target proteins. The constructed genes were inserted into the pBI121 binary expression vector [16] to obtain pBI-Endo H and pBI- PNGase F. Plasmids were then introduced into the *Agrobacterium tumefaciens* strain AGL1. Transformed *A*. *tumefaciens* strain AGL1 was grown in BBL medium (10 g/L soy hydrolysate, 5 g/L yeast extract, 5 g/L NaCl, and 50 mg/L kanamaycin) overnight at 28°C and then introduced by manual infiltration by syringe into 6-7-week-old *N*. *benthamiana* plants as a mixture with *A*. *tumefaciens* strain AGL1 carrying a silencing suppressor at a ratio of OD600 0.9 and 0.1. Leaf tissue was harvested at 6 dpi and homogenized in three volumes of extraction buffer. After centrifugation at 13,000 g for 20 min, diluted samples were run on SDS–PAGE prior to Western blotting. The expression levels of Endo H and PNGase F were calculated by quantifying protein levels from western blots by Syngene gel imaging system using GeneTools Software. *A*. *tumefaciens* strain AGL1and P19 (plant suppressor of RNA silencing) plasmid [17] was kindly provided by Professor Sophien Kamoun (The Sainsbury Laboratory, Norwich, UK). A wild type *N*. *benthamiana* was grown on soil in a greenhouse of Akdeniz University.

Purification of plant produced Endo H was performed by anti-FLAG affinity chromatography using anti-DYKDDDDK affinity gel (Cat. No. 651503, BioLegend). For purification, 7 g of frozen leaves was ground in 20 mL PBS buffer (1XPBS, 150 mM NaCI) using a mortar and a pestle. Plant debris was removed by filtration through Miracloth followed by centrifugation at 20,000 g for 25 minutes and then filtered through a 0.45 μm syringe filter (Millipore). An anti-FLAG affinity column was prepared according to the manufacturer’s instructions. Twenty one milliliters of a clear supernatant were loaded into 1.0 ml resin column equilibrated with PBS buffer. The column was washed with 10 volumes of PBS buffer. Bound proteins were eluted using 200 mM Glycine, 150 mM NaCl, pH 2.2 into tubes containing 2.0 M Tris solution to neutralize. After estimating total protein content using the Bradford assay method, fractions containing protein were combined and concentrated with a 10k MWCO Amicon Ultra 0.5 mL concentrator to a final volume of 0.1 mL and buffer exchanged against PBS. Concentrated samples were analyzed by SDS-PAGE and stored at -80°C until use.

To assess the deglycosylating activity of the recombinant plant produced Endo H *in vitro*, 50 ng of plant produced Endo H, purified by anti-FLAG affinity chromatography was combined with 10 μg of plant produced PA83, purified by IMAC, in 1X GlycoBuffer 3 (New England Biolabs) and incubated at 37°C for 1 h. To compare the deglycosylation by plant produced Endo H with commercial Endo H or PNGase F, PA83 protein was incubated with commercial Endo H (Cat. No. P0702S, New England Biolabs) and PNGase F (Cat. No. P0704S, New England Biolabs) according to the manufacturer’s instructions for the non-denaturing reaction conditions. For deglycosylation of PA83 by commercial Endo H or PNGase F, 10 μg PA83 proteins were combined with 1 μl of Endo H (0.5 μg, 500 units) or 1 μl of PNGase F (500 units), in 1X GlycoBuffer 3 or GlycoBuffer 2 (New England Biolabs), respectively, and then incubated at 37°C for 1 hour. 2 μg or 100 ng PA83 protein from each sample was analyzed by SDS-PAGE or Western blot analysis, respectively. Proteins were probed with the protective antigen antibody (BAP0101, Cat. No. ab1988, Abcam). The image was taken using high sensitive GeneGnome XRQ Chemiluminescence imaging system (Syngene, A Division of Synoptics Ltd).

### Co-expression of Endo H with *B*. *anthracis* PA, Pfs48/45 and Pfs48/45-10C, and evaluation of its deglycosylating activity *in vivo*

The sequences of *B*. *anthracis* PA (amino acids 30–764, GenBank accession number AAA22637) and Pfs48/45 (amino acids 28–401, GenBank accession number EU366251, the full-length form of Pfs48⁄45, 16 cysteine residues and seven putative glycosylation sites) were optimized for expression in *N*. *benthamiana* plants and synthesized by GENEART AG (Thermo Fisher Scientific). Pfs48/45-10C (amino acids 159–401, GenBank accession number EU366251, truncated form of Pfs48/45, contains I, II, III epitops, 10 cycteines and five potential N-glycosylation sites) [15, 18] was amplified by PCR using a Pfs48/45 gene, synthesized by GENEART AG, as a DNA template. PR-1a signal peptide (MGFVLFSQLPSFLLVSTLLLFLVISHSCRA) was added to the N-terminus of all genes. In addition, the KDEL sequence (the ER retention signal) and the FLAG epitope (for Pfs48/45 and Pfs48/45-10C, the affinity purification tag) or His tag (for PA83) were added to the C-terminus. The resulting sequences were inserted into the pBI121 and pEAQ [19] binary expression vectors to obtain pBI-PA83, pEAQ-Pfs48/45 and pEAQ-Pfs48/45-10C. To co-express Endo H or PNGase F with *B*. *anthracis* PA, pBI-EndoH/pBI-PA83 or pBI-PNGase F/pBI-PA83 constructs were used for infiltration. To co-express Endo H with Pfs48/45 or Pfs48/45-10C, pBI-EndoH/ pEAQ-Pfs48/45 or pBI-EndoH/ pEAQ-Pfs48/45-10C, constructs were used for infiltration. Similarly, to co-express PNGase F with Pfs48/45 or Pfs48/45-10C, pBI-PNGase F/pEAQ-Pfs48/45 and pBI-PNGase F/pEAQ-Pfs48/45-10C were used for infiltration. Since the pEAQ vector enables both the gene of interest and the suppressor of silencing to be expressed from a single plasmid, the pEAQ constructs (pEAQ-Pfs48/45 or pEAQ-Pfs48/45-10C) were infiltrated into 6–7 week old *N*. *benthamiana* plants without a mixture of *A*. *tumefaciens* AGL1 strain carrying a silencing suppressor. In order to achieve complete deglycosylation of PA83, Pfs48/45 and Pfs48/45-10C proteins *in vivo*, plant infiltration was optimized with the use of different ratios of *A*. *tumefaciens* strain AGL1expressing Endo H, PNGase F or target proteins. Leaf samples were taken at 5 dpi (for PA83) or 7dpi (for Pfs48/45 or Pfs48/45-10C) and were homogenized in three volumes of extraction buffer. After centrifugation at 13,000 g, samples were diluted 2-fold in the SDS sample buffer. 10 μl liters of samples were run on SDS–PAGE followed by Western blotting. PA83 bands were detected using the anti-4xHis tag mAb (BioLegend, Cat. no. 652502); Ps48/45, Endo H or PNGase F bands were detected using the purified anti-DYKDDDDK tag antibody (anti-FLAG antibody) (BioLegend, Cat. No. 637301). The pEAQ binary expression vector was kindly provided by Dr. George P. Lomonossoff (John Innes Centre, Biological Chemistry Department).

### Purification of glycosylated and non-glycosylated plant produced Pfs48/45 and Pfs48/45-10C proteins from *N*. *benthamiana*

Pfs48/45 was engineered and expressed with a C-terminal FLAG epitop to facilitate detection and purification of recombinant proteins. Purification of plant produced glycosylated and non-glycosylated Pfs48/45 variants from *N*. *benthamiana* were performed following a similar procedure using Anti-DYKDDDDK Affinity Gel (Cat. No. 651503, BioLegend). For purification, 10 g of frozen leaves from each sample was ground in TBS buffer (20 mM Tris Buffer, 150 mM NaCI, pH 7.5) using a mortar and a pestle and then followed the same procedure as described above for the purification of plant produced Endo H. Concentrated samples were analyzed by SDS-PAGE and stored at -80°C until use.

Pfs48/45-10C was engineered and expressed with a C-terminal 6-His tag as described above. Plant produced deglycosylated Pfs48/45-10C, was purified by immobilized metal ion affinity chromatography (IMAC) using HisPur™ Ni-NTA resin (Cat. No. 88221, Thermo Fisher Scientific). For purification, twenty grams of frozen plant leaves infiltrated with Pfs48/45-10C were ground in 20 mM sodium phosphate, 300 mM sodium chloride, 10 mM imidazole, pH 7.4 (extraction buffer) and the extract was centrifugated for 25 minutes at 4°C at 20,000 g, and the supernatant was filtered by using 0.45 μm Nalgene™ filter (Thermo Fisher Scientific, USA). The filtered supernatant was loaded onto a disposable polypropylene column (Pierce) with HisPur™ Ni-NTA resin equilibrated with 20 mM sodium phosphate, 300 mM sodium chloride, 10 mM imidazole, pH 7.4, by gravity-flow chromatography. The column was washed with 8 column volumes (CV) of wash buffer (20 mM sodium phosphate, 300 mM sodium chloride, 25 mM imidazole; pH 7.4) and eluted with 8 CV of elution buffer (20 mM sodium phosphate, 300 mM sodium chloride, 250 mM imidazole; pH 7.4). Elution fractions were collected and protein concentrations in the eluted fractions were determined by Bradford assay. The combined fractions were concentrated and buffer exchanged against PBS and concentrated with a Millipore concentrator (10K MWCO) to a final volume of 0.25 ml. The concentrated protein was stored at –80°C until use.

*In vivo* deglycosylated Pfs48/45-10C proteins, produced by Endo H and PNGase F co-expression, were purified from 10 grams of infiltrated frozen *N*. *benthamiana* leaves in a similar manner as plant produced glycosylated Pfs48/45-10C.

### Purification of glycosylated and non-glycosylated plant produced PA83 proteins from *N*. *benthamiana*

Glycosylated, Endo H or PNGase F *in vivo* deglycosylated PA83 variants were purified from *N*. *benthamiana* leaves as described previously (4). Fifty grams of frozen plant leaves infiltrated with glycosylated, Endo H or PNGase F deglycosylated PA83 proteins were ground in a phosphate extraction buffer. The supernatant was passed through Miracloth and centrifuged at 20,000 g for 25 minutes, and then passed through 0.45 μm filter (Millipore).The filtered supernatant was passed through the HisTrap FF 5 mL column (GE, Cat. No. 17-5255-01), previously equilibrated with 50 mM sodium phosphate, containing 0.5 M NaCl, 20 mM imidazole, pH 7.5, at a flow rate of 5.0 mL/min using a peristaltic pump. After washing the column with 10 CV of equilibration buffer. bond proteins were eluted with 10 CV of elution buffer (50 mM sodium phosphate, 0.5 M NaCl, 150 mM imidazole, pH 7.5). Elution fractions were collected and protein concentrations in the eluted fractions were determined by Bradford assay. The combined fractions were concentrated and buffer exchanged against PBS and concentrated with a Millipore concentrator (10K MWCO) to a final volume of 2.5 ml and stored overnight in –20°C. After two days, the concentrated protein was thawed and centrifuged at 20,000 x g for 4 minutes at 4°C. Fine powdered ammonium sulfate was added to the supernatant to a final concentration of 1.0 M, and the supernatant was loaded into HiTrap phenyl HP 1 mL column (GE, Cat. No. 17-1351-01), equilibrated with PBS, 1.0 M ammonium sulfate at a rate of 0.8 mL/min. After washing the column with 8 CV of equilibration buffer, proteins were eluted with 8 CV of elution buffer (1XPBS, 0.6 M ammonium sulfate). Total protein content in elution fractions was estimated using the Bradford assay, and then fractions with protein content were combined, buffer exchanged against PBS. and concentrated to 0.45 ml. The concentrated proteins were stored in –80°C until use.

### Culture of hybridoma cells secreting anti-Pfs48/45 mAb

MRA-26, mouse hybridoma cells secreting mAb anti-Pfs48/45 (IIC5B-10-1, anti-*Plasmodium falciparum* 48/45-kDa Gamete Surface Protein) was obtained from Malaria Research and Reference Reagent Resource Center (MR4). Before plating the cells, the frozen vial was thawed immediately in a water bath at 37°C. Before opening, the outside of the vial was wiped with 70% ethanol. Cells were then were resuspended in RPMI medium including 5% FBS, 1% Pen-Strep, 1% NEAA, 1& GlutaMax and β-ME and centrifuged at 2700g for 5 min at 37°C in a 15ml conical tubes. Supernatant including freezing medium was discarded and cells were dissolved in the same medium described above. Totally 12 ml medium including cells were plated in a T25 flask and placed in an incubator vertically. Every three days cells were passaged in 1:5 ratio. Hybridoma cells were cultured as described above for nearly two weeks. During the last passage RPMI medium without serum was added on serum grown cells to a final FBS concentration of 2%. Reduced serum medium was separated from cells with a quick spin and filter sterilized through 0.4 micron membrane. Nearly 500 ml filter sterilized medium and 5x10^6^ cells crude/vial were kept in -80°C separately. Anti-Pfs48/45 mAb was purified from the medium using Protein A affinity column.

### SDS-PAGE, native PAGE and western blot analysis

Protein samples were separated on 10% SDS–PAGE, transferred onto a polyvinylidene fluoride membrane (Millipore, Billerica, MA) and blocked with 0.5% I-block (Applied Biosystems, Carlsbad, CA). His tagged proteins were detected using **a** purfied mouse anti-His Tag antibody (Cat. no. 652502, BioLegend). The FLAG tagged recombinant proteins were detected using anti-FLAG mAbs (Cat. No.637301, BioLegend). A PA83 protein was detected either with the purified anti-His Tag antibody (Cat. No. 652502, BioLegend) or anti-Bacillus anthracis protective antigen antibody BAP0101 (Cat. No. ab1988, Abcam). The membranes were then washed with 1xPBS (Phosphate-buffered saline) containing 0.1% Tween- 20 (PBS-T) to remove an excess primary antibody and then labeled with an anti-mouse horseradish peroxidase (HRP)-conjugated secondary antibody (Cat. No. ab98790, Abcam) or anti-rabbit horseradish peroxidase (HRP)-conjugated secondary antibody (Cat. No. ab97051, Abcam). Reducing samples were prepared in 5X Laemli Buffer to obtain final concentrations of 100 mM Tris, 2% SDS, 20% glycerol, 4% β-mercaptoethanol, pH 6.8. Non-reducing gel and samples were prepared in the same manner with no SDS and reducing agent added. Native PAGE is performed using native sample and running buffers without denaturants or SDS. Proteins separated by Native PAGE were probed using anti-His tag, anti-FLAG and anti-Pfs48/45 mAb(MRA-26) antibodies. Signal generation was achieved with a chemiluminescent substrate (SuperSignal West Pico, Thermo Fisher Scientific, Grand Island, NY). The images were taken using the GeneSnap software on a GeneGnome and quantified using the Gene Tools software (Syngene Bioimaging, UK).

### Glycan detection analysis

The presence of glycans in plant-produced, partially purified Pfs48/45-10C and PA83 variants (glycosylated, Endo H or PNGase F *in vivo* deglycosylated) were detected by Pro-Q Emerald 300 glycoprotein staining. About 0.25μg of the plant-produced PA83 and Pfs48/45-10C variants (glycosylated and *in vivo* deglycosylated forms) were run on 10% SDS–PAGE followed by the detection of glycans in the gel using the Pro-Q Emerald Glycoprotein Stain Kit according to the manufacturer’s protocol (Pro-Q® Emerald 300 Glycoprotein Gel Stain Kit, with SYPRO® Ruby Protein Gel Stain, P21855, Life technologies, Molecular Probes). Stained proteins were visualized using UV illumination.

### Stability assessment

Plant produced glycosylated and deglycosylated variants of PA83 were diluted to 1.0 mg/mL, and aliquoted into low-binding, polypropylene Eppendorf tubes, and then incubated at 4°C for 72 hours, and at 37°C for 1, 2, 4, 8, 16 and 24 hours. After incubation, samples were mixed with SDS with SDS loading dye (5X). Samples were then stored at -20°C until used for SDS–PAGE analysis. For calculating the degradation of PA83 variants, coomassie blue stained protein bands of each sample from SDS-PAGE were quantified by Syngene gel imaging system using GeneTools Software.

### Deglycosylation efficiency of plant produced Endo H and PNGase F

Anti-Pfs48/45 mAb was purified from the medium using protein A affinity column.

To study the efficiency of deglycosylation of plant produced Endo H and PNGase F *in vivo*, the Pfs48/45-10C, Pfs48/45 and PA83 constructs were co-infiltrated with the Endo H or PNGase F at various OD600 of Agrobacteria carrying Endo H, PNGase F and target genes. The infiltration ratios were 1.0: 0; 0.9: 0.1; 0.8: 0.2; 0.7: 0.3; 0.6: 0.4 and 0.5: 0.5 for the target proteins and deglycosylating enzymes, respectively. The efficiency of deglycosylation was evaluated by Western blot analysis, using size reduction as an indicator of glycan removal. The blots were probed with the anti-His tag or anti-FLAG.

To study the deglycosylation efficiency of plant produced Endo H and PNGase F *in vitro*, both Endo H and PNGase F recombinant enzymes were freshly purified from 10 g of frozen leaves using the procedure described above for the purification of Endo H. 10 μg of plant produced PA83 was incubated at 37°C for 1 h with different amounts (0, 25, 100, 200, 400, 800 ng) of plant produced Endo H or PNGase F, in GlycoBuffer 3 (New England Biolabs) or GlycoBuffer 2 (New England Biolabs), respectively. To compare the deglycosylation efficiency of plant produced Endo H with commercial Endo H or PNGase F, PA83 protein was incubated with commercial Endo H (Cat. no. P0702S, New England Biolabs) and PNGase F (Cat. no. P0704S, New England Biolabs) according to the manufacturer’s instructions for the non-denaturing reaction conditions.

## Results

### Expression, purification and *in vitro* activity assessment of plant produced Endo H

**Fig 2:**
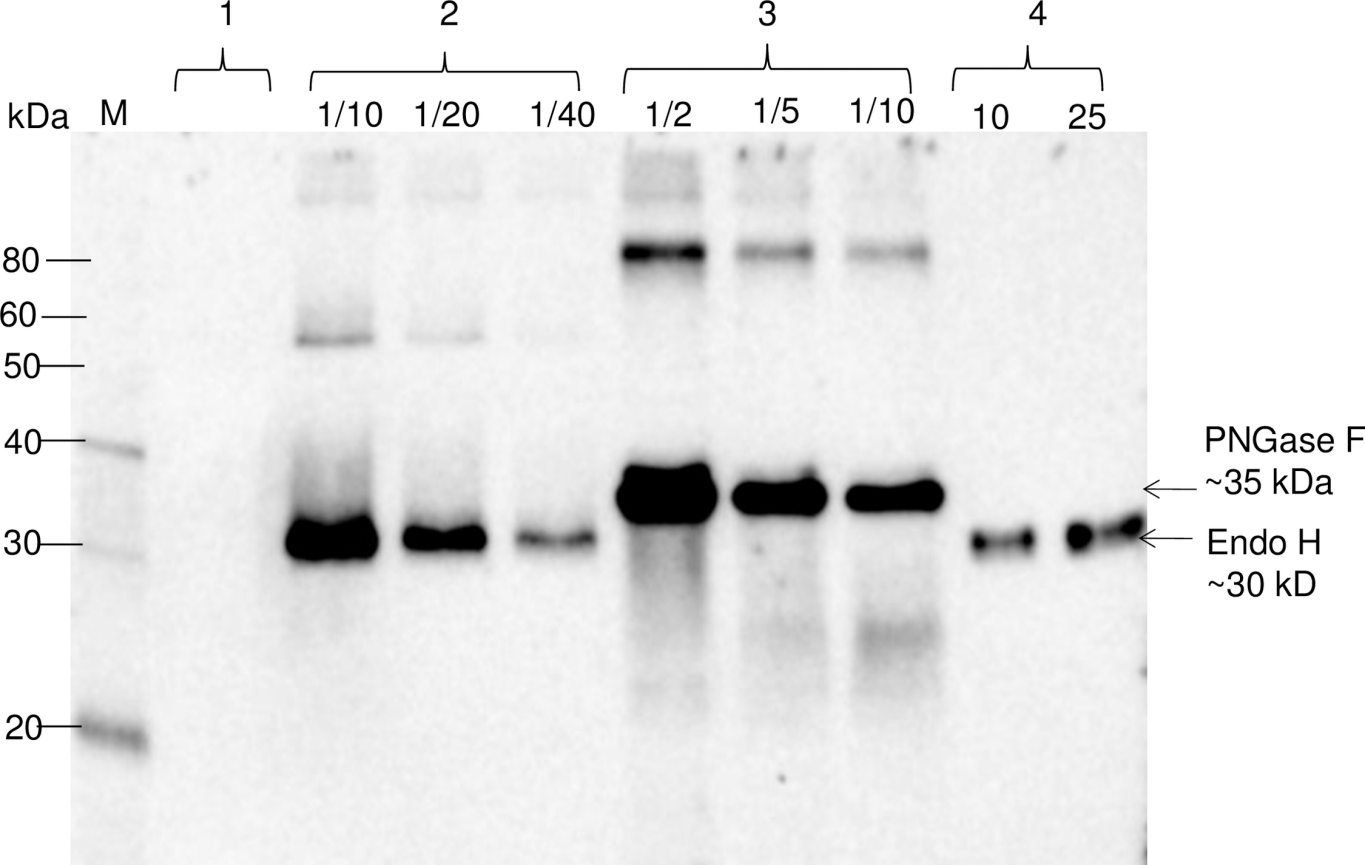
Western blot analysis of bacterial Endo H or PNGase F produced in *Nicotiana benthamiana* plants. *N*. *benthamiana* plants were infiltrated with pBI-Endo H or pBI-PNGase F constructs to produce Endo H or PNGase F. Lanes: 1-crude extract prepared from control plant; 2- crude extract prepared from plant infiltrated with bacterial Endo H (pBI-Endo H) and 10, 20 and 40 fold diluted samples were loaded into gel; 3- crude extract prepared from plant infiltrated with bacterial PNGase F (pBI-PNGase F),and 2, 5 or 10 fold diluted samples were loaded into gel; 4- purified plant produced Endo H used as a standard protein; 10 or 25 ng were loaded into gel. M: MagicMark XP Western Protein Standard (ThermoFisher Scientific).

The bacterial Endo H gene was optimized for the expression in *N*. *benthamiana* plants, cloned into the pBI121 vector and expressed in *N*. *benthamiana* plants as described in Materials and Methods. In addition, a PNGase F gene was also cloned, constructed, and co-expressed with target proteins to compare the deglycosylation efficiency of these two deglycosylating enzymes in plants. The expression of Endo H and PNGase F proteins in *N*. *benthamiana* was confirmed by the Western blot analysis using an anti-FLAG antibody (Fig 2). As shown in Fig 2, plant produced recombinant Endo H (~30 kDa) migrates faster than PNGase F protein (~35 kDa) on the gel. The expression levels of Endo and PNGase F in *N*. *benthamiana* plant were estimated by running different dilutions of crude extract proteins on Western blot, together with the plant produced, purified Endo H protein (see below) as a standard. The expression level of Endo H was higher than 170 mg/kg of fresh leaf biomass. The expression level of PNGase F was approximately 135 mg/kg of fresh leaf biomass. Notably, *N*. *benthamiana* plants expressing Endo H remained healthy at 7 days post-infiltration (dpi) with no visible symptom development when co-expression of target proteins reached the highest level (data not shown).

**Fig 3:**
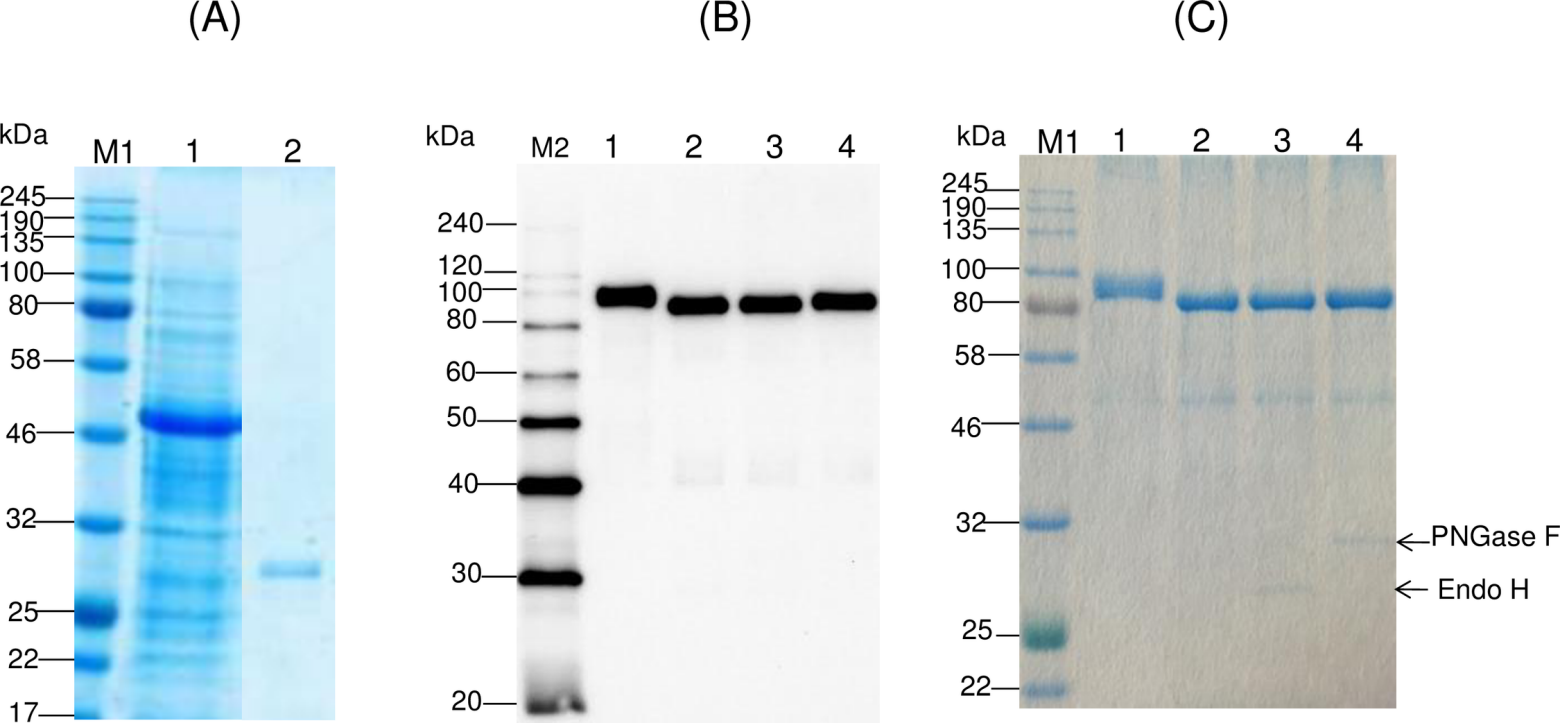
SDS-PAGE analysis of plant produced, purified bacterial Endo H from *N*. *benthamiana* plants and evaluation of its deglycosylating activity *in vitro*. (A) SDS-PAGE analysis of purified plant produced Endo H from *N*. *benthamiana* plant. Lanes: 1-About 20 μg of the crude supernatant was loaded; 2–0.75 μg purified Endo H was loaded. (B), (C) Western blot or SDS-PAGE analysis of PA83 protein incubated with either plant produced Endo H or commercial Endo H or commercial PNGase F. Lanes: 1- plant produced PA83; 2- plant produced PA83 was treated with the plant produced Endo H; 3- plant produced PA83 was treated with the commercial Endo H; 4- plant produced PA83 was treated with the commercial PNGase F. 100 ng or 2 μg PA83 protein samples were loaded in each lane in Western blot and SDS-PAGE, respectively. M1: color prestained protein standard (New England Biolabs); M2: MagicMark XP Western Protein Standard (ThermoFisher Scientific). Arrows in C indicates migration of commercial Endo H and PNGase F.

Purification of plant produced Endo H was performed using anti-FLAG chromatography as described in Materials and Methods. Purified plant produced Endo H was highly homogeneous (Fig 3A, lane 2). To evaluate the deglycosylating activity of the recombinant plant-produced Endo H *in vitro*, the purified plant-produced Endo H enzyme was incubated with plant produced glycosylated, purified PA83 protein. SDS-PAGE and Western blot analysis confirmed that plant produced Endo H was able to deglycosylate PA83 protein *in vitro* (Fig 3B and 3C, lane 2). Similar results were obtained when PA83 proteins were incubated with commercial Endo H or PNGase F (New England Biolabs) (Fig 3B and 3C, lanes 3 and 4).

### *In vivo* deglycosylation of recombinant *B*. *anthracis* PA, Pfs48/45 and Pfs48/45-10C in *N*. *benthamiana* plants by co-expressing Endo H

**Fig 4:**
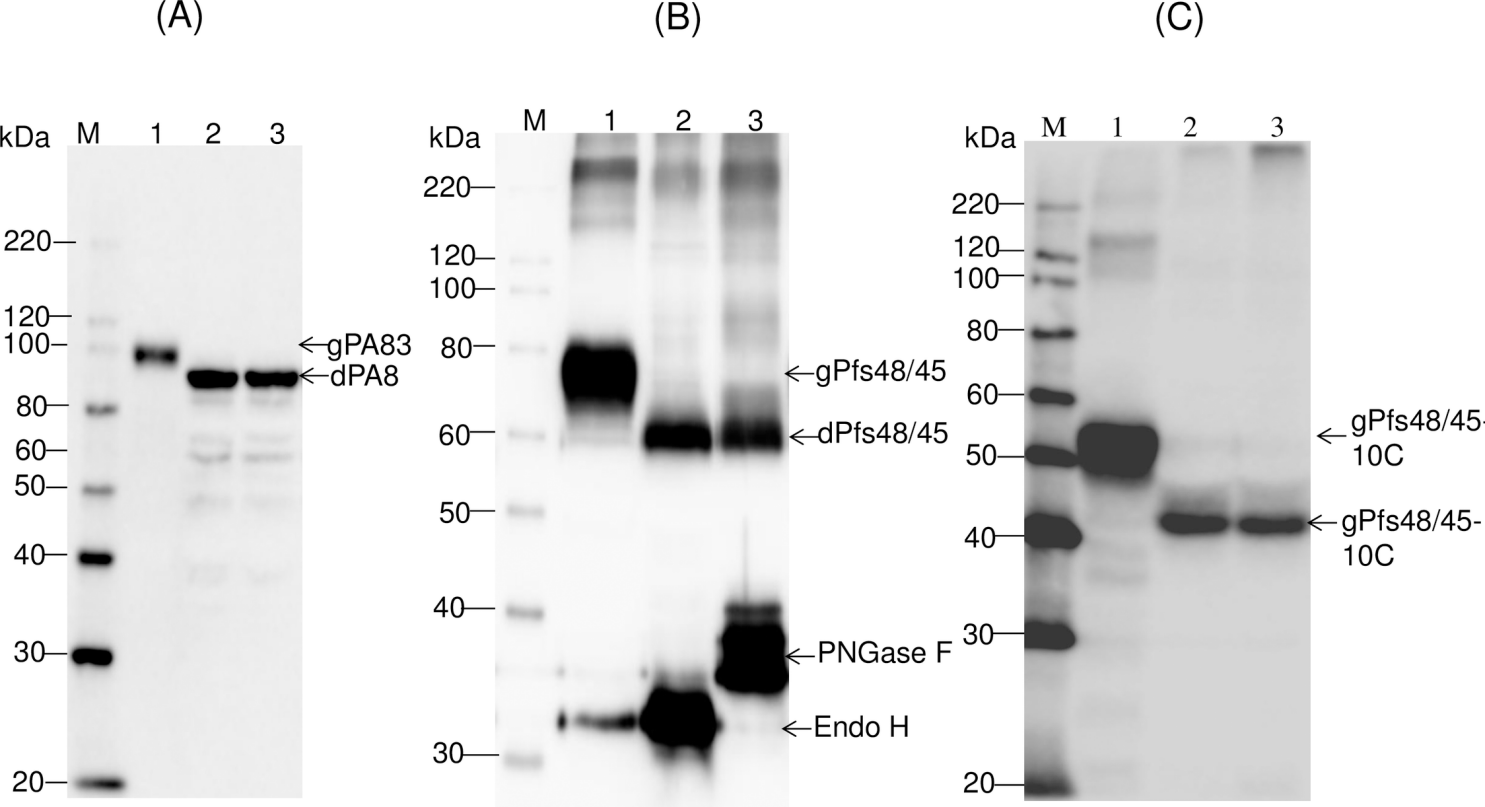
Western blot analysis of co-expression *Bacillus anthracis* PA83 (A), Pfs48/45 (B) and Pfs48/45-10C with bacterial Endo H or PNGase F in *N*. *benthamiana* plants. (A) Western blot analysis of co-expression of PA83. Lanes: 1- *N*. *benthamiana* plant was infiltrated with pBI-PA83 construct, for the production of glycosylated PA83, 2,3- *N*. *benthamiana* plants were infiltrated with combinations of the pBI-Endo H/pBI-PA83 or pBI-PNGase F/pBI-PA83 constructs, for the production of Endo H (2) or PNGase F (3) deglycosylated PA83 proteins. (B) Western blot analysis of co-expression of Pfs48/45. Lanes: 1-*N*. *benthamiana* plant was infiltrated with pEAQ-Pfs48/45 construct for the production of glycosylated Pfs48/45;2,3- *N*. *benthamiana* plants were infiltrated with combinations of the pBI-Endo H/pEAQ-Pfs48/45 or pBI-PNGase F/pEAQ-Pfs48/45constructs for the production of Endo H (2) and PNGase F (3) deglycosylated Pfs48/45 proteins. (C) Western blot analysis of co-expression of Pfs48/45-10C. Lanes: 1- *N*. *benthamiana* plant was infiltrated with pEAQ-Pfs48/45-10C construct for the production of glycosylated Pfs48/45-10C; 2,3- *N*. *benthamiana* plants were infiltrated with combinations of the pBI-Endo H/pEAQ-Pfs48/45 or pBI-PNGase F/pEAQ-Pfs48/45constructs for the production of Endo H (2) and PNGase F (3) deglycosylated Pfs48/45-10C proteins. gPA83- glycosylated PA83; dPA83- deglycosylated PA83; gPfs48/45: glycosylated Pfs48/45; dPfs48/45: deglycosylated Pfs48/45; gPfs48/45-10C: glycosylated Pfs48/45-10C; dPfs48/45-10C: deglycosylated Pfs48/45-10C.M: MagicMark XP Western Protein Standard (ThermoFisher Scientific). PA83 proteins were detected using the anti-Bacillus anthracis protective antigen antibody BAP0101 (Cat. No. ab1988, Abcam); Ps48/45, Endo H or PNGase F proteins were detected using the anti-FLAG antibody (BioLegend). Pfs48/45-10C protein was detected using the purified anti-His Tag antibody (Cat. No. 652502, BioLegend).

To evaluate *in vivo* cleavage of N-linked oligosaccharides decorating PA83, Endo H and PA83 were transiently co-expressed in *N*. *benthamiana* plants via co-agroinfiltration with both, pBI-PA83 or pBI-Endo H constructs. A bacterial PNGase F was also transiently co-expressed in *N*. *benthamiana* plants via co-agroinfiltration with both pBI-PA83 and pBI-PNGase F constructs, to compare with the Endo H deglycosylation. It should be noted that PA83 of *B*. *anthracis* is not a glycoprotein, however, its sequence has six potential glycosylation sites and it has been shown to be glycosylated when expressed in *N*. *benthamiana* plants [2,4]. The Western blot analysis performed at 5 dpi demonstrated a shift in the mobility of PA83 co-expressed with Endo H (Fig 4A, lane 2), suggesting protein deglycosylation. In addition, as shown by SDS–PAGE and Western blot analysis (proteins were probed with the protective antigen antibody, BAP0101, Cat. No. ab1988, Abcam), co-expression with Endo H led to the accumulation of PA83 that was similar in size to that of the *in vivo* deglycosylated molecule by bacterial PNGase F (Fig 4A, lane 3) indicating that PA83 was enzymatically deglycosylated. It should be noted that smaller bands were observed in Western blot for deglycosylated PA83 proteins, produced *in vivo* by co-expressing with bacterial Endo H or PNGase F (Fig 4A, lane 2 and 3). Since these deglycosylated PA83 samples were prepared from crude protein extracts, these results suggest that deglycosylation probably renders the protein more susceptible to proteases.

This approach was further tested by co-expressing the enzyme with Pfs48/45 and Pfs48/45-10C. Pfs48/45 is one of the leading candidates for transmission-blocking (TB) vaccine development. It was shown that the native Pfs48⁄45 protein does not contain N-linked glycans [18]. Pfs48⁄45 protein has seven putative N-linked glycosylation sites and these sites can be aberrantly glycosylated when the protein is expressed in any of the available eukaryotic hosts [2]. To evaluate *in vivo* cleavage of N-linked oligosaccharides decorating Pfs48/45, bacterial Endo H and Pfs48/45 protein of *Plasmodium falciparum* were transiently co-expressed in *N*. *benthamiana* plants via co-agroinfiltration with pBI-Endo H and pEAQ-Pfs48/45 constructs. As shown by Western blot analysis, co-expression with Endo H led to the accumulation of Pfs48/45 with molecular mass of about ~60kDa (Fig 4B, lane 2), which is similar in size to that of the *in vitro* deglycosylated molecule by PNGase F (Fig 4B, lane 3), indicating that Pfs48/45 was enzymatically deglycosylated. Deglycosylation of Pfs48/45-10C protein by Endo H was also demonstrated by Western blot (Fig 4C, lane 2) analysis. These results demonstrate that co-expression of Pfs48/45-10C (~50kDa) with Endo H led to the accumulation of Pfs48/45-10C with molecular mass of about ~40kDa (Fig 4C, lane 2), similar in size to that of the *in vitro* deglycosylated form by PNGase F (Fig 4C, lane 3). Deglycosylation of Pfs48/45 or Pfs48/45-10C by Endo H or PNGase F was also demonstrated by SDS-PAGE (see below).

Taken together, the results demonstrate that Endo H successfully cleaved N-linked glycans from PA83 and Pfs48/45 proteins and plant produced Endo H is enzymatically active both *in vivo*, and *in vitro*. As we described above, Endo H enzyme cleaves high mannose and hybrid N-linked glycans, however, does not cleave the complex N-linked glycans from asparagine-linked glycoproteins, and therefore the strategy would work only if target proteins are retained in the ER.

### Glycan detection analysis of glycosylated and deglycosylated PA83 variants

**Fig 5:**
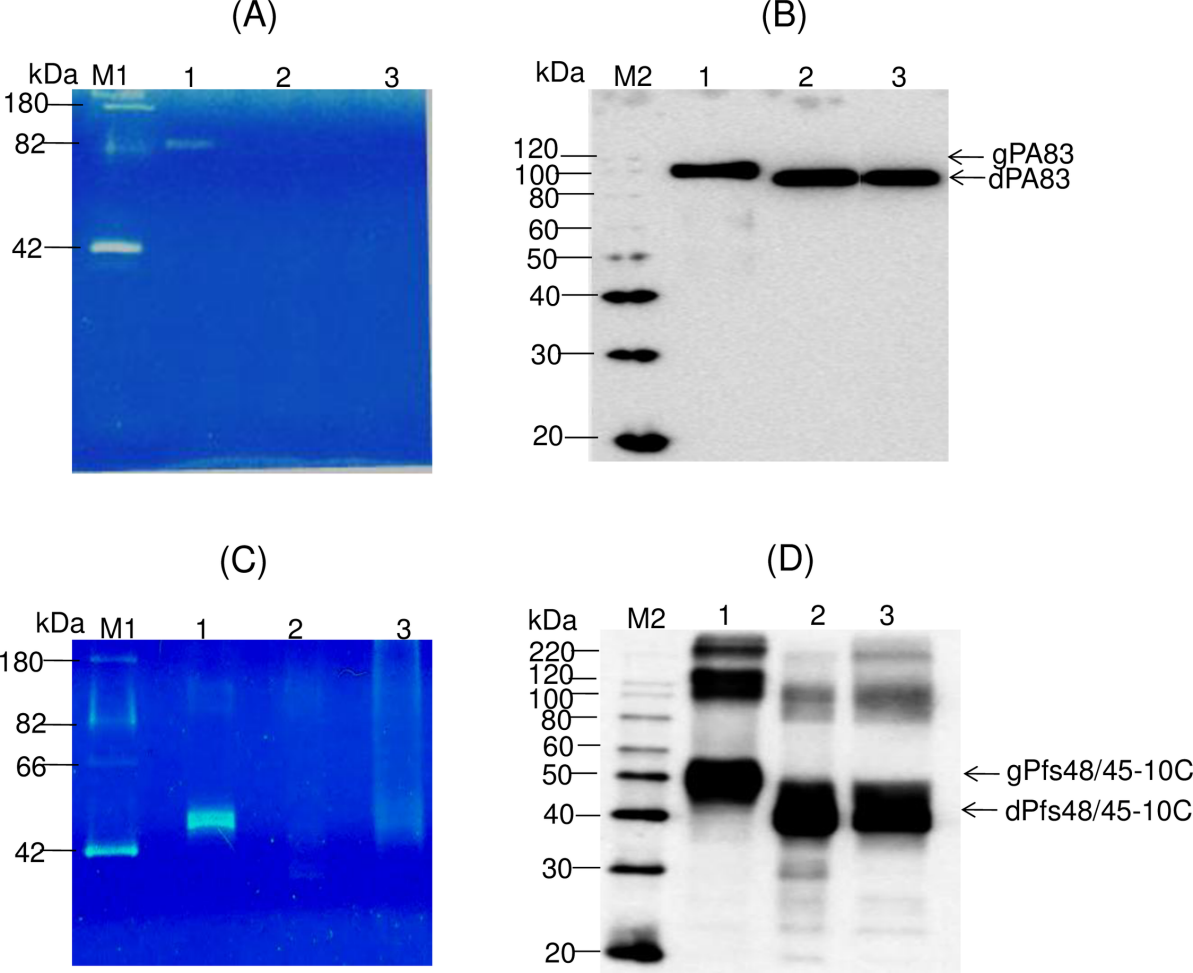
Glycan detection and Western blot analysis of glycosylated and *in vivo* deglycosylated PA83 and Pfs48/45-10C variants. (A), (C) 0.25 μg of protein from each sample was run on a 10% SDS-PAGE followed by in-gel glycan detection using the Pro-Q Emerald 300 glycoprotein staining kit. Stained proteins were visualized by UV illumination. (B), (D) Western blot analysis of the same samples using anti-His Tag antibody (BioLegend). (A), (B) Lanes: 1 –plant produced glycosylated PA83; 2– deglycosylated PA83, produced by *in vivo* deglycosylation of PA83, co-expressed with Endo H; 3– deglycosylated PA83, produced by *in vivo* deglycosylation of PA83, co-expressed with PNGase F. (C), (D) Lines: 1 –plant produced glycosylated Pfs48/45-10C; 2–deglycosylated Pfs48/45-10C, produced by *in vivo* deglycosylation of Pfs48/45-10C, co-expressed with Endo H; 3– deglycosylated Pfs48/45-10C, produced by *in vivo* deglycosylation of Pfs48/45-10C, co-expressed with Endo H. M1: CandyCane glycoprotein molecular weight standards (Molecular Probes), 250 ng of each protein per lane. M2: MagicMark XP Western Protein Standard (ThermoFisher Scientific). Indications of gPA83, dPA83, gPfs48/45-10C, dPfs48/45-10C are the same as shown in Fig 4.

To demonstrate the *in vivo* deglycosylation of target proteins by Endo H, we also performed the glycan detection analysis of glycosylated and deglycosylated PA83 and Pfs48/45-10C variants as described in Materials and Methods. As shown in Fig 5, the glycan was only detected in plant produced glycosylated PA83 (Fig 5A, lane 1) and Pfs48/45-10C (Fig 5C, lane 1). Fig 5B and 5D show the Western blot analysis of the same samples (1/5 fold diluted) probed with an anti-His tag antibody.

### Recognition of plant produced, Endo H or PNGase F deglycosylated Pfs48/45 or Pfs48/45-10C proteins with conformational specific Pfs48/45 monoclonal antibody

**Fig 6:**
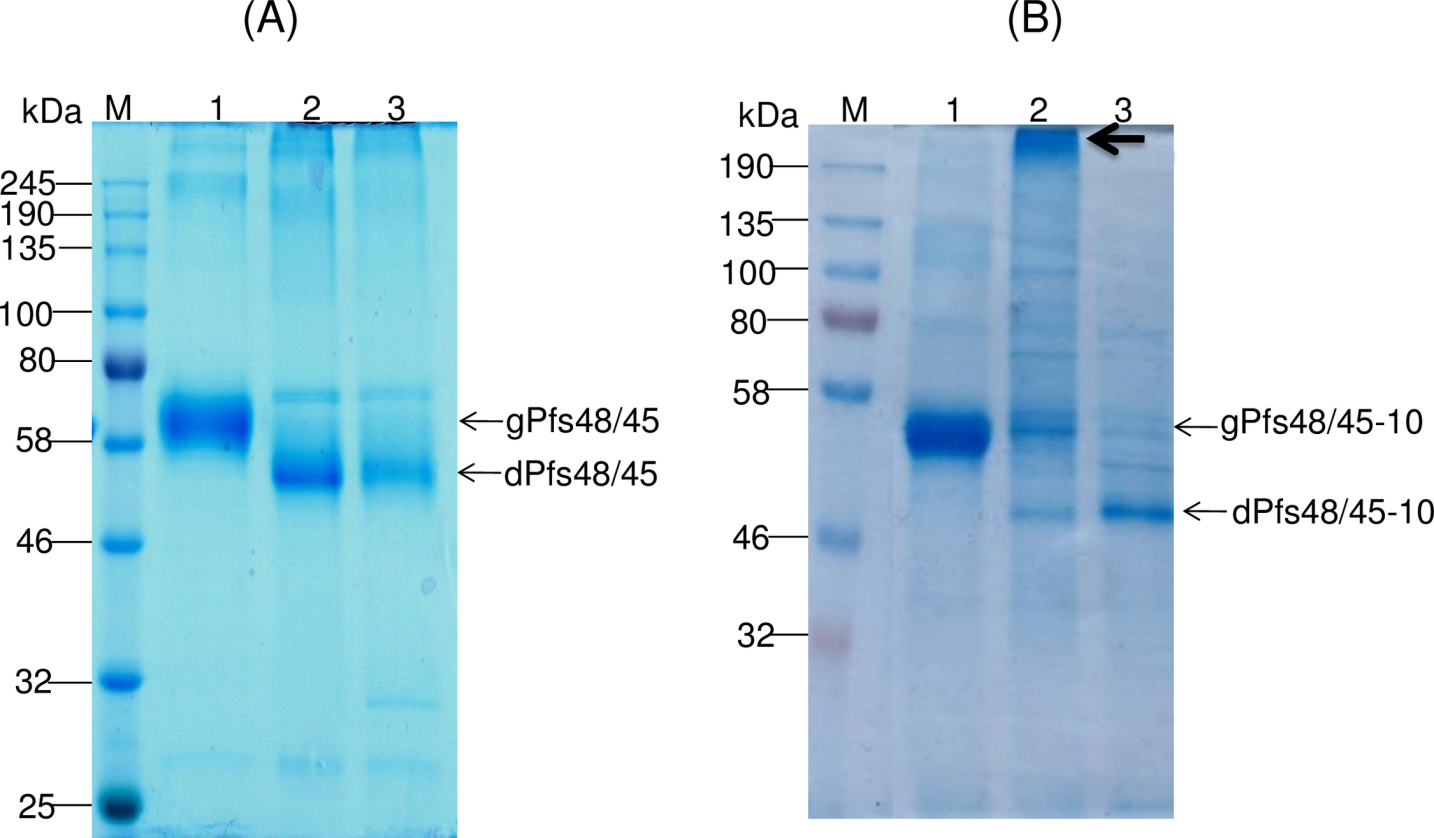
SDS-PAGE analysis of purified plant produced Pfs48/45 variants. Lanes were loaded with ~1.0 μg (A) or ~2.0 μg (B) per lane for glycosylated, Endo H or PNGase F *in vivo* deglycosylated plant produced Pfs48/45proteins. (A) Lanes: 1- glycosylated Pfs48/45; 2- deglycosylated Pfs48/45, produced by *in vivo* deglycosylation of Pfs48/45, co-expressed with Endo H; 3- deglycosylated Pfs48/45, produced by *in vivo* deglycosylation of Pfs48/45, co-expressed with PNGase F. (B) Lanes: 1- glycosylated Pfs48/45-10C; 2- deglycosylated Pfs48/45-10C, produced by *in vivo* deglycosylation of Pfs48/45-10C,co-expressed with PNGase F; 3- deglycosylated Pfs48/45, produced by *in vivo* deglycosylation of Pfs48/45-10C, co-expressed with PNGase F. Indications of gPfs48/45, dPfs48/45, gPfs48/45-10C and dPfs48/45-10C are the same as shown in Fig 5. M: color prestained protein standard (New England Biolabs). Arrow in B (indicating lane:2) shows aggregation of deglycosylated Pfs48/45-10C protein, produced by *in vivo* deglycosylation of Pfs48/45 co-expressed with PNGase F.

**Fig 7:**
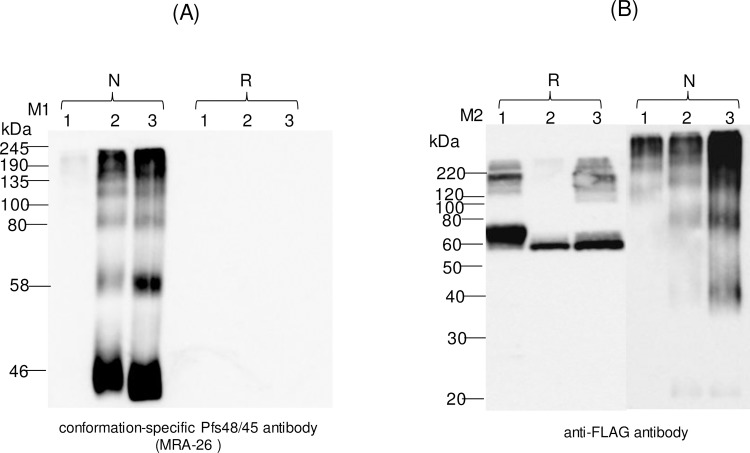
Western blot analysis of Pfs45/48 variants using the MRA-26 antibody, a conformational specific Pfs48/45 mAb. Western blot analysis of Pfs45/48 variants using the MRA-26 antibody compared with the anti-FLAG antibody (A) Native PAGE followed by Western blot analysis of Pfs45/48 variants using the MRA-26 antibody. (B) Samples that were analyzed by Native PAGE, were also analyzed on SDS-PAGE, and proteins were probed with anti-FLAG antibody. Lanes: 1- glycosylated Pfs48/45; 2- deglycosylated Pfs48/45, produced by *in vivo* deglycosylation of Pfs48/45, co-expressed with Endo H; 3- deglycosylated Pfs48/45, produced by *in vivo* deglycosylation of Pfs48/45, co-expressed with PNGase F. Reduced (R) and non-reduced (N) samples were prepared as described in the Materials and Methods. M1: color prestained protein standard (New England Biolabs); M2: MagicMark XP Western Protein Standard (ThermoFisher Scientific). Western blot using a conformation-specific anti-Pfs48/45 antibody showed that reduction of the plant produced Pfs48/45 recombinant protein prevents recognition by antibody when compared with Western analysis using a FLAG antibody.

**Fig 8:**
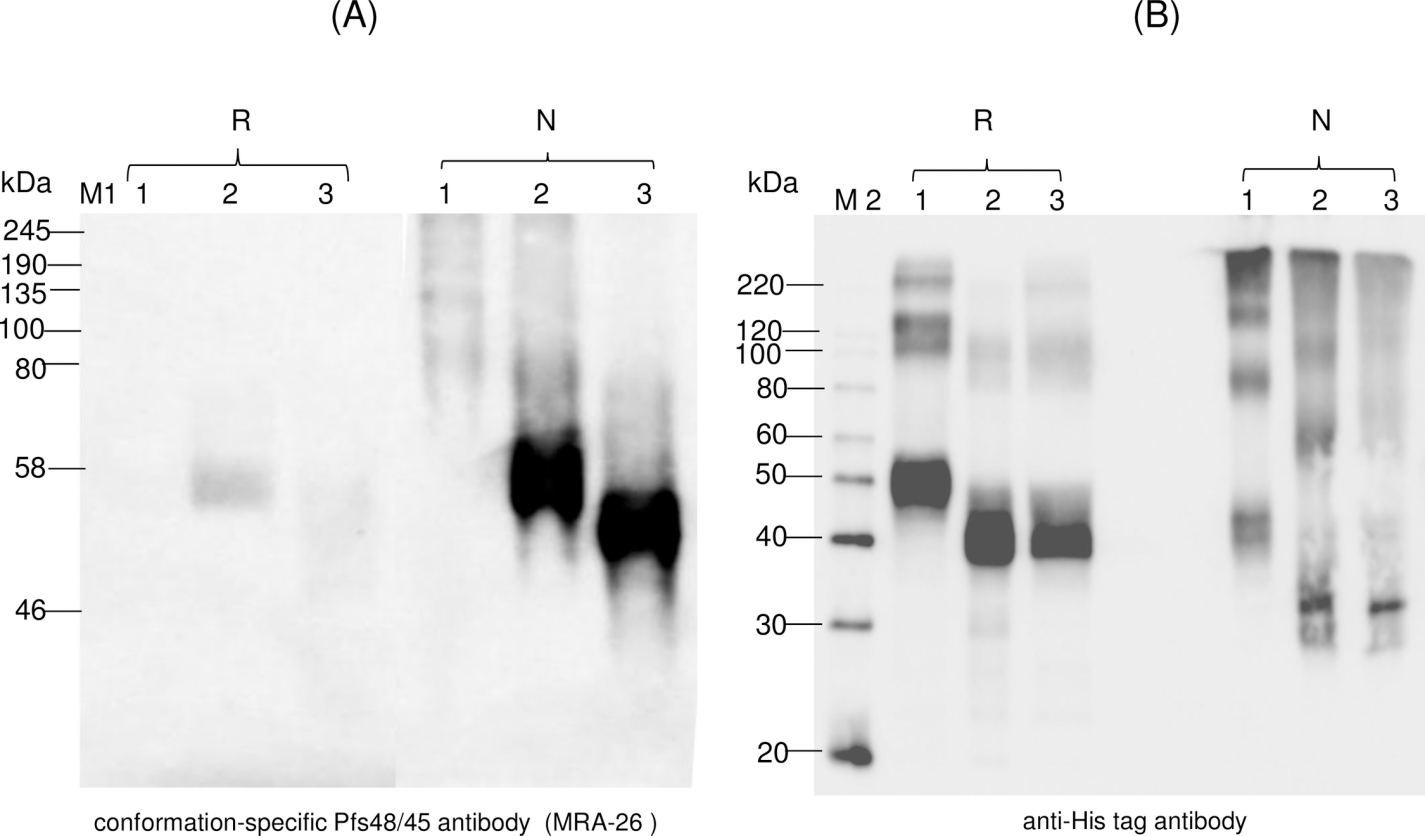
Western blot analysis of Pfs45/48-10C variants using MRA-26 antibody, a conformational specific Pfs48/45 mAb. Western blot analysis of Pfs45/48-10C variants using the MRA-26 antibody compared with the anti-His tag antibody. (A) Native PAGE followed by Western blot analysis of Pfs45/48-10C variants using the MRA-26 antibody. (B) Samples that were analyzed by Native PAGE, were also analyzed on SDS-PAGE, and proteins were probed with anti-His tag antibody. Lanes: 1- glycosylated Pfs48/45-10C; 2- deglycosylated Pfs48/45-10C, produced by *in vivo* deglycosylation of Pfs48/45, co-expressed with Endo H; 3- deglycosylated Pfs48/45-10C, produced by *in vivo* deglycosylation of Pfs48/45, co-expressed with PNGase F. Reduced (R) and non-reduced (N) samples were prepared as described in the Materials and Methods. M1: color prestained protein standard (New England Biolabs); M2: MagicMark XP Western Protein Standard (ThermoFisher Scientific). Western blot using a conformation-specific anti-Pfs48/45 antibody showed that reduction of the plant produced Pfs48/45 recombinant protein prevents recognition by antibody when compared with Western analysis using a His Tag antibody.

A monoclonal IIC5-B10 antibody [20] is a conformational specific Pfs48/45 monoclonal antibody (mAb). MRA-26 mouse hybridoma cells secreting mAb anti-Pfs48/45 were cultured to secrete mouse mAb against Pfs48/45 (mAb IIC5-B10 or MRA-26) in the medium. In order to test recognition of plant produced Endo H or PNGase F *in vivo* deglycosylated Pfs48/45 or Pfs48/45-10C variants with conformational specific Pfs48/45 monoclonal antibody, plant produced Pfs48/45 (FLAG tagged) or Pfs48/45-10C proteins (His tagged) were purified using anti-DYKDDDDK Affinity Gel or HisPur™ Ni-NTA resin, respectively, as described in Materials and Methods. Fig 6 shows SDS-PAGE analysis of plant produced, partially purified glycosylated, Endo H or PNGase F *in vivo* deglycosylated Pfs48/45 (A) or Pfs48/45-10C (B) proteins. Equivalent amounts of reduced and non-reduced samples of purified proteins from each sample (proteins were quantified using the gene tools software, Syngene Bioimaging) were run on SDS-PAGE or Native PAGE, transferred to PVDF membrane and proteins on the membrane were probed with anti-FLAG, anti-His tag antibody or a conformational specific MRA-26 antibody. Fig 7 shows the Western blot of Pfs48/45 variants using the MRA-26 antibody compared with anti-FLAG antibody. As can be seen in Fig 7, anti-FLAG antibody recognizes the glycosylated, Endo H and PNGase F *in vivo* deglycosylated proteins in both reduced and non-reduced samples. However, conformational specific MRA-26 antibody recognizes plant produced Endo H and PNGase F *in vivo* deglycosylated Pfs48/45 proteins better than the glycosylated counterpart, suggesting correct folding of *in vivo* Endo H or PNGase F deglycosylated Pfs48/45 proteins. Similar results were obtained with Pfs48/45-10C protein. Fig 8 shows that anti-His tag antibody recognizes glycosylated, Endo H and PNGase F *in vivo* deglycosylated Pfs48/45-10C proteins in both reduced and non-reduced samples. The fact that a conformational specific MRA-26 antibody does not recognize or weakly recognizes the aberrantly glycosylated Pfs48/45-10 in non-reduced samples, but strongly recognizes plant produced Endo H and PNGase F *in vivo* deglycosylated Pfs48/45-10 proteins in non-reduced samples, suggests correct folding of plant produced, *in vivo* Endo H or PNGase F deglycosylated Pfs48/45-10C proteins. It is noteworthy that MRA-26 antibody does not recognize plant produced Endo H and PNGase F *in vivo* deglycosylated Pfs48/45 (Fig 7) or Pfs48/45-10C (Fig 8) proteins in reduced samples, suggesting that conformational specific Pfs48/45 mAb binding is highly sensitive to reduction. Taken together, these data suggest that Endo H or PNGase F *in vivo* deglycosylated forms of Pfs48/45 or Pfs48/45-10C proteins contain epitopes present on native Pfs48/45.

### Purification of glycosylated and Endo H or PNGase F deglycosylated PA83 proteins and stability assessments of different variants of PA83

**Fig 9:**
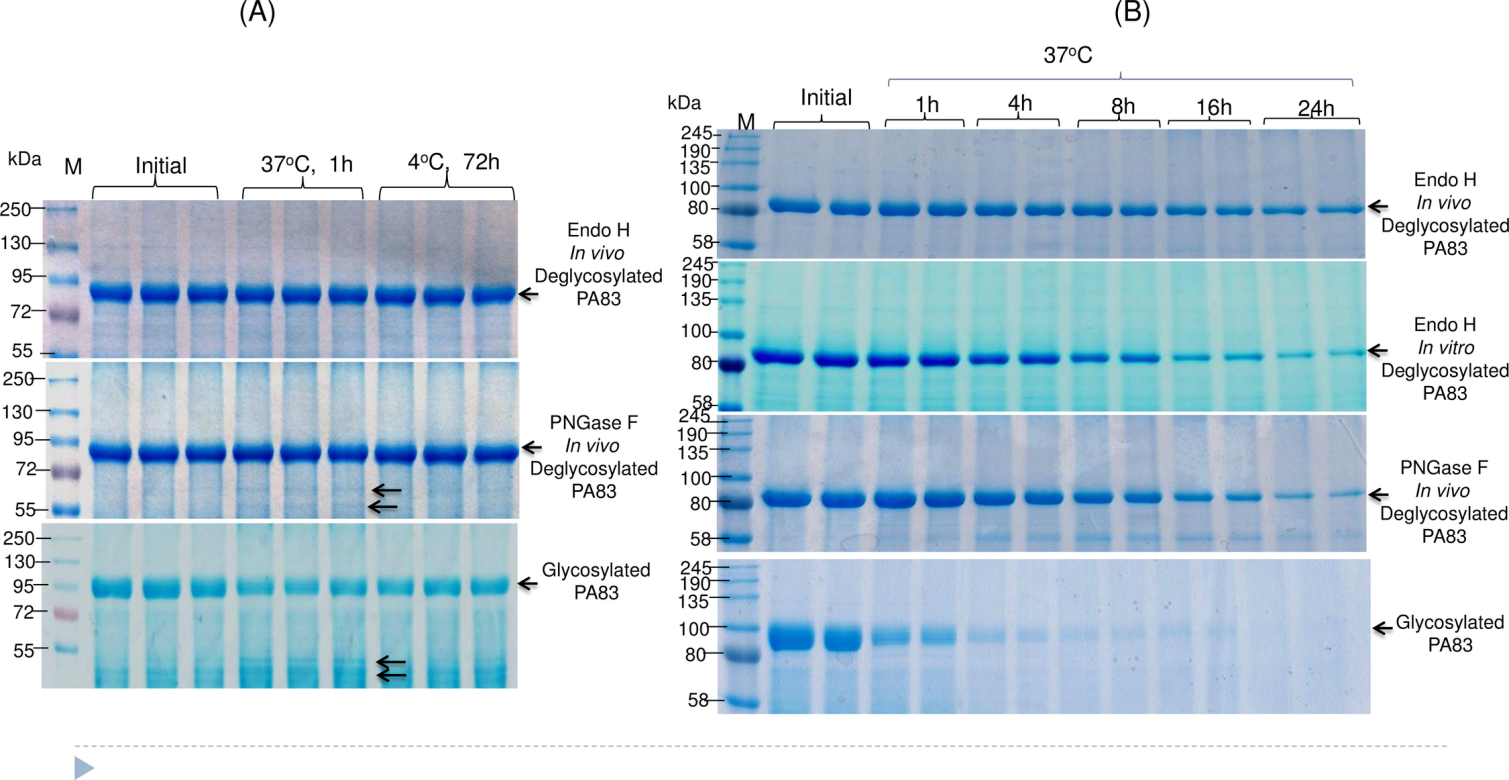
Study of stability of glycosylated and deglycosylated PA83 variants using SDS-PAGE analysis. Plant produced, glycosylated PA83, and *in vivo* Endo H or PNGase F deglycosylated forms of PA83 were purified as described in Materials and Methods. (A) The purified plant produced PA83 variants were stored for 1 hour at 37°C or for 72 hours at 4°C and analyzed by SDS-PAGE. Lanes were loaded with ~8.0 μg per lane for glycosylated, Endo H or PNGase F *in vivo* deglycosylated plant produced PA83 proteins. (B) Purified, plant produced, glycosylated PA83, and *in vivo* deglycosylated (co-expressed with Endo H or PNGase F) and *in vitro* deglycosylated (by commercial Endo H) proteins were incubated at 37°C for 1, 4, 8, 16 and 24 hours, and analyzed in SDS-PAGE. Lanes were loaded with ~5.0 μg per lane for each sample. M- color prestained protein standard (New England Biolabs).

Plant produced, glycosylated PA83, and *in vivo* deglycosylated forms of PA83, co-expressed with Endo H or PNGase F, were purified using HisTrap FF followed by Hydrophobic interaction chromatography (HiTrap phenyl HP) as described in Materials and Method. As shown by SDS–PAGE and Coomassie staining, the purified deglycosylated forms of PA83 proteins were highly homogeneous. The stability of plant produced glycosylated and *in vivo* deglycosylated forms of PA83 were examined after incubation at 37°C for 1 hour or at 4°C for 72 hours, using a method similar to those described previously[4]. Analysis by SDS-PAGE showed the glycosylated and PNGase F deglycosylated plant produced PA83s degraded by more than 66% and 17%, respectively, after incubation at 37°C for 1 hour, whereas Endo H deglycosylated PA83 showed approximately 10% degradation (Fig 9A).

The stability of PA83 co-expressed with Endo H and PNGase F was further examined after incubation at 37°C for a longer period of time: 1, 4, 8, 16 and 24 hours. Analysis by SDS-PAGE showed that glycosylated PA83 degraded 71.70%, 91.40%, 95.80%, 95.90% and 100% for 1, 4, 8, 16 and 24 hours, respectively. Degradation of *in vivo* PNGase F glycosylated PA83 for 1, 4, 8, 16 and 24 hours were 7.20%, 25.70%, 40.70%, 66.20% and 83.50%, respectively. Endo H deglycosylated PA83 showed better stability at 37°C and degraded 5.50%, 13.20%, 27.60%, 35.60% and 49.30% for 1, 4, 8, 16, and 24 hours, respectively. Notably, a PA83, deglycosylated by commercial Endo H *in vitro*, degraded 8.96%, 33.28%, 55.23%, 78.87% and 88.67% for 1, 4, 8, 16, and 24 hours, respectively, more rapidly than the Endo H *in vivo* deglycosylated form (Fig 9B).

*In vitro* half-life (time to 50% remaining) of glycosylated, PNGase F *in vivo* deglycosylated, Endo H *in vitro* deglycosylated and *in vivo* Endo H deglycosylated forms of PA83 were calculated by analyzing coomassie stained protein bands on SDS-PAGE, which were 33.96 min, 10.75 hours, 8.33 hours and 24 hours respectively. These results demonstrate that the plant produced Endo H *in vivo* deglycosylated form of PA83 appeared to be more stable compared to Endo H *in vitro* or PNGase F *in vivo* deglycosylated counterparts at elevated temperatures.

### Deglycosylation efficiency of Endo H versus PNGase F

**Fig 10:**
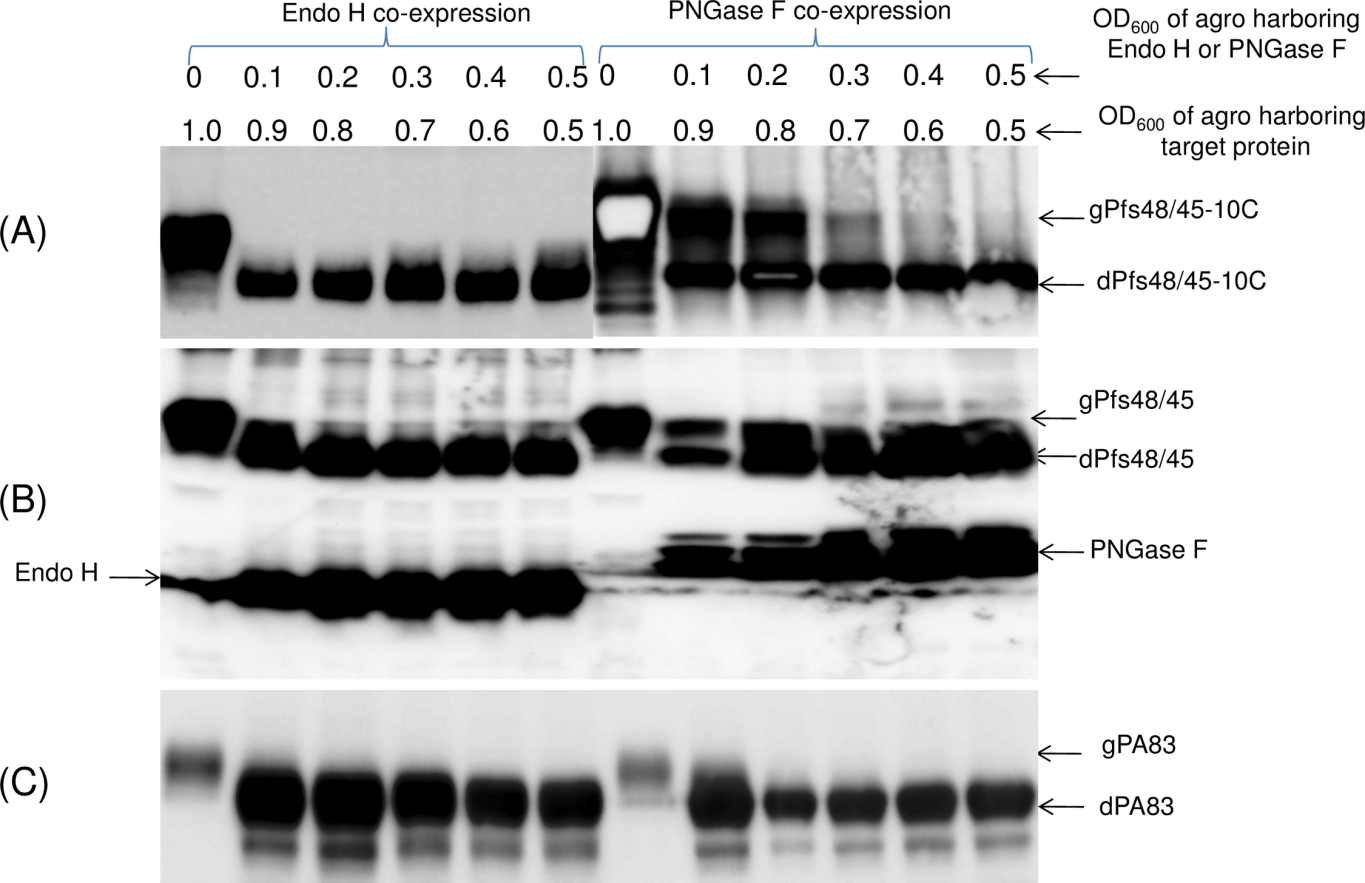
Evaluation of the deglycosylation efficiency of Pfs48/45-10C by Endo H or PNGase F *in vivo*. Pfs48/45-10C (A), Pfs48/45 (B) and PA83 (C) were co-expressed with Endo H or PNGase F at different ratios of OD600 of Agrobacteria carrying Endo H, PNGase F and target genes, as indicated. The efficiency of deglycosylation of target proteins by Endo H or PNGase F was evaluated by Western blot analysis. Size reduction of Pfs48/45-10C, Pfs48/45 and PA83 show as an indicator of glycan removal. Proteins were probed with the anti-4xHis Tag mAb (BioLegend) (A,C) or anti-FLAG antibody (B). Indications of proteins in figures are the same as shown above.

When *A*. *tumefaciens* strain AGL1harboring Pfs48/45-10C and Endo H genes were infiltrated into *N*. *benthamiana* plant at 0.9: 0.1 OD (optical density, OD600) ratio, respectively, a complete deglycosylation of Pfs48/45-10C was observed (Fig 10A, left panel). However, complete deglycosylation of Pfs48/45-10C protein by PNGase F was observed at higher than 0.5 OD ratio (0.5:0.5 OD ratio of *A*. *tumefaciens* strain AGL1carrying PNGase F or Pfs48/45-10C, respectively) of agrobacterium carrying PNGase F (Fig 10A, Right panel). Similar results were observed with other protein targets, such as Pfs48/45 proteins (Fig 10B) and PA83 (Fig 10C).

**Fig 11:**
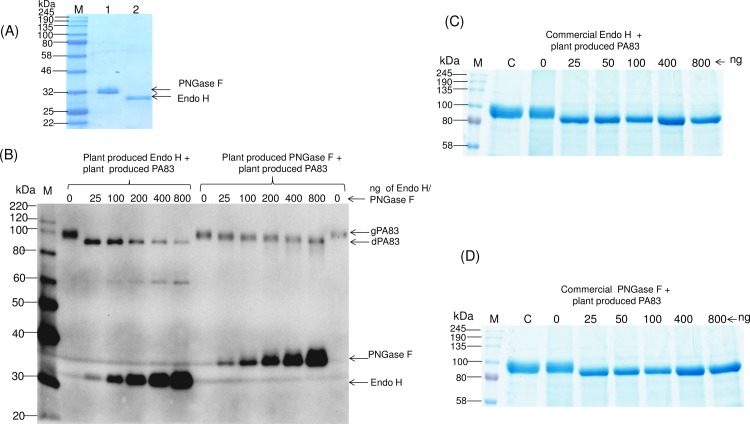
Study of the deglycosylation efficiency of plant produced Endo-H against PNGase F *in vitro*. (A) SDS-PAGE analysis of purified plant produced PNGase F and Endo H. Lanes were loaded with 1.0 μg per lane. 1-plant produced PNGase F; 2- plant produced Endo H; M-color prestained protein standard (New England Biolabs). (B) Plant produced PA83 was incubated with different amounts (0, 25, 100, 200, 400, 800 ng) of plant produced Endo H or PNGase F, as indicated. After incubation, proteins were analyzed by SDS-PAGE followed by Western blot analysis. Proteins were detected using a mixture of anti-His Tag antibody to detect His tagged PA83 and anti-FLAG antibody to detect FLAG-tagged Endo H or PNGase F. M: MagicMark XP Western Protein Standard (ThermoFisher Scientific). (C) Plant produced PA83 was incubated at 37°C for 1 h with different amounts (0, 25, 50, 400 and 800 ngs) of commercial Endo H, as indicated. Lanes, C- PA83 protein was kept at 4°C for 1 h; M-color prestained protein standard (New England Biolabs). (D) Plant produced PA83 was incubated at 37°C for 1 h with different amounts (0, 25, 50, 400 and 800 ngs) of commercial PNGase F, as indicated. Lanes, C- PA83 protein was kept at 4°C for 1 h; M- color prestained protein standard (New England Biolabs).

The deglycosylation efficiency of plant produced Endo H in comparison with plant produced PNGase F, commercial Endo H or PNGase F, was further monitored *in vitro*. Different amounts of plant produced (0, 25, 100, 200, 400 and 800 ng) or commercial Endo H and PNGase F (0, 25, 50, 100, 400 and 800 ng) were incubated with 10 μg of the plant produced, purified PA83 substrate at 37°C for 1 hour. Plant produced Endo H and PNGase F proteins were purified at the same time using anti-FLAG affinity chromatography as described in Materials and Methods. After purification, the plant producing PNGase F (Fig 11A, lane 1) and Endo H (Fig 11A, lane 2) proteins were very pure, as analyzed by SDS-PAGE. The deglycosylation efficiency of plant produced Endo H and PNGase F *in vitro* was monitored and verified by Western blot analysis. As can be seen from Fig 11B (Left panel), a complete deglycosylation of the plant produced PA83 substrate was observed when 25 ng plant produced Endo H was incubated with 10 μg of PA83 protein; however, complete deglycosylation of plant produced PA83 was observed at 800 ng of the plant produced PNGase F (Fig 11B, right panel). Thus, these results demonstrate that the deglycosylation efficiency of plant produced Endo H is greater than that of plant produced PNGase F. Notably, the efficiency of the deglycosylation of plant produced Endo H was similar to those of commercial Endo H (Fig 11C) and commercial PNGase F (Fig 11D); a complete deglycosylation of the plant produced PA83 substrate was observed when 25 ng of either commercial Endo H or commercial PNGase F were incubated with 10 μg of PA83 protein.

## Discussion

Numerous studies in recent years demonstrated that plant based transient expression system has high expression capacity and provides safe, fast, inexpensive production of valuable recombinant proteins of interest within a short time frame [21–25]. However, if recombinant proteins are aberrantly N-glycosylated during expression in plants, this system may not be suitable for production of those proteins in plants. Therefore, for the expression of various proteins with different post-translational modification status, more flexible approaches are needed to preserve their native sequence and biological function. In the present study, we describe *in vivo* deglycosylation of recombinant N-glycosylated proteins as a result of their transient co-expression with the bacterial enzyme Endo H in plants. Our results showed that plant produced recombinant Endo H is fully active *in vivo* and *in vitro*, and successfully cleaved N-linked glycans from malaria vaccine candidates Pfs48/45 and Pfs48/45-10C, as well as from PA83 of *B*. *anthracis*. In addition, we demonstrate that enzymatic deglycosylation of target proteins *in vivo* by Endo H had a positive impact on the stability in that the deglycosylated PA83 molecule produced by Endo H appeared to be more stable than the PNGase F deglycosylated counterpart, which may greatly increase the life and duration of vaccine storage and thereby reduce the cost of the vaccine considerably. Thus, Endo H deglycosylated plant produced PA83 of *B*. *anthracis* might be another potential vaccine candidate against anthrax.

Pfs48/45 is one of the leading candidates for TB vaccine development and plays a key role in parasite fertilization. The protein is present on the surface of gametocytes, gametes, and zygotes. A challenge to developing a vaccine based on Pfs48/45 is the production of correctly folded recombinant proteins [26]. Pfs48/45 is a complex cysteine rich (16 cysteines involved in disulfide bond formation) membrane anchored protein with seven predicted N-linked glycosylation sites. Correct formation of disulfide bridges is critically important for proper folding of many cysteine-rich proteins, including Pfs48/45. It was demonstrated that immunization with Pfs48/45 protein results in the production of malaria TB antibodies. Our previous study demonstrated that epitope-specific mAbs I, III, and V recognized the PNGase F deglycosylated form of Pfs48/45 2- to 6-fold better than the glycosylated form [2]. In this study, we demonstrate that, in contrast to the glycosylated form, plant produced *in vivo* Endo H deglycosylated Pfs48/45 and Pfs48/45-10 were recognized by conformational specific Pfs48/45 monoclonal antibody, a known TB antibody, in a manner similar to its PNGase F deglycosylated counterpart. It should be noted that the IIC5B-10-1 mAbs is known to target antigens of TB immunity on gametes of *P*. *falciparum* [27,28]. In addition, a conformation-specific antibody, IIC5-10 recognizes epitope III (between the residues 302–327) on Pfs48/45 when the protein is folded, and the correct pattern of disulfide bonds is present [27–32]. Thus, Endo H or PNGase F *in vivo* deglycosylated forms of Pfs48/45 and Pfs48/45-10C contain epitopes present in native Pfs48/45. Recombinant Pfs48/45-10C has been previously expressed in the *Escherichia coli* expression system; however, after chemical-induced refolding, only 10–20% was correctly folded [33]. Much improved refolding (~90%) of the truncated Pfs48/45-10C was obtained through co-expression of *E*. *coli* chaperones that elicited functional TB antibodies in immunized mice [15]. Our results showed that the expression levels, solubility and purification yields of Endo H deglycosylated Pfs48/45 or Pfs48/45-10C proteins were higher than those deglycosylated by PNGase F. In addition, as can be seen in the Fig 6, purified PNGase F deglycosylated Pfs48/45-10C tends to aggregate, especially at higher concentrations. Thus, plant produced Endo H *in vivo* deglycosylated Pfs48/45 and Pfs48/45-10C antigens have a potential for the development of a Pfs48/45-based TB malaria vaccine.

Taken together, the data supports the fact that the Endo H co-expression strategy provides another opportunity to produce important vaccine antigens, including a vaccine against anthrax, TB vaccines against malaria, and also therapeutic proteins, antibodies, and recombinant enzymes for therapeutic use and industrial applications. Development of stable transgenic plants expressing Endo H for use as an expression host, could be a useful tool. As we described above, *N*. *benthamiana* plants expressing Endo H remained healthy at 7 dpi with no visible symptom development when co-expression of target proteins reached the highest level. Because of the transient nature of expression and the brief time span, the effect of Endo H on the endogenous protein folding and extracellular secretion was not significant. However, in plants, most proteins of the extracellular compartment and the endomembrane system are glycosylated and their N-linked glycosylation has a great impact on their biological functions [34]. Based on our data (above); since Endo H possesses high deglycosylation efficiency *in vivo*, stable transgenic plants expressing even a low level of Endo H may have detrimental effects on the growth or development of plants caused by expression of Endo H. At this point, development of stable transgenic plants expressing Endo H using an inducible system, could be a more appropriate tool.

Thus, Endo H *in vivo* deglycosylation technology could be expected to have potential applications in molecular farming, in the pharmaceuticals field to produce subunit vaccines, therapeutic proteins, and antibodies in the deglycosylated forms. This study can be also important for the production of industrial enzymes, especially bacterially originated enzymes, in eukaryotic expression system for increasing bioenergy, and biofuel yield, as well as improving food quality, especially by producing native additives. The expression level of plant produced recombinant Endo H was higher than 170 mg/kg leaf biomass. The yield could be enhanced after optimization of the expression conditions of Endo H in plants. Endo H has not been previously expressed in plants, and therefore, plant expression systems may be an ideal platform for the economical, high-level expression of recombinant endotoxin-free Endo H with high enzymatic activity. Finally, this approach can be applied to different PTMs for producing proteins with desired activity in heterologous systems.

In summary, this and our previous studies collectively demonstrate that enzymatic deglycosylation of target proteins *in vivo* has the potential to become a robust strategy for the production of non-glycosylated proteins in plants. Notably, the properties (activity, immunogenicity, etc.) of proteins produced by *in vivo* glycosylation differ from *in vitro* deglycosylated counterparts. Although *in vivo* and *in vitro* deglycosylated PA83 proteins have theoretically identical amino acid sequences, the lethal toxin neutralizing activity and immunogenicity of plant produced *in vivo* deglycosylated PA83 was greater than those of *in vitro* deglycosylated PA83 [4]. In this study, our results demonstrate that the plant produced Endo H *in vivo* deglycosylated form of PA83 appeared to be more stable compared to the Endo H *in vitro* deglycosylated form at elevated temperatures, suggesting potential differences in protein folding of the *in vivo* and *in vitro* deglycosylated forms.

## References

[1] Gomord V, Faye L. (2004) Posttranslational modification of therapeutic proteins in plants. Curr Opin Plant Biol 7: 171–181. doi: 10.1016/j.pbi.2004.01.015 15003218 10.1016/j.pbi.2004.01.015

[2] Mamedov T, Ghosh A, Jones RM, Mett V, Farrance CE, Musiychuk K, et al (2012) Production of non glycosylated recombinant proteins in *Nicotiana benthamiana* plants by co-expressing bacterial PNGase F. Plant Biotechnol J 10:773–782. doi: 10.1111/j.1467-7652.2012.00694.x . 22520228 10.1111/j.1467-7652.2012.00694.x

[3] Mamedov T, Yusibov V. (2013) *In vivo* deglycosylation of recombinant proteins in plants by co-expression with bacterial PNGase F. Bioengineered 4(5):338–42. doi: 10.4161/bioe.23449 23328084 10.4161/bioe.23449 PMC3813534

[4] Mamedov T, Chichester JA, Jones RM, Ghosh A, Coffin MV, Herschbach K, et al (2016) Production of functionally active and immunogenic non-glycosylated protective antigen from Bacillus anthracis in Nicotiana benthamiana by co-expression with peptide-N-glycosidase F (PNGase F) of Flavobacterium meningosepticum. PLoS One 21;11(4):e0153956 doi: 10.1371/journal.pone.0153956 27101370 10.1371/journal.pone.0153956 PMC4839623

[5] Hägglund P, Bunkenborg J, Elortza F, Jensen ON, Roepstorff P. (2004) A new strategy for identification of N-glycosylated proteins and unambiguous assignment of their glycosylation sites using HILIC enrichment and partial deglycosylation. J Proteome Res 3:556–66. 15253437 10.1021/pr034112b

[6] Muramatsu T. (1971) Demonstration of an endo-glycosidase acting on a glycoprotein. Journal of Biological Chemistry 246(17): 5535–5537. 4108054

[7] Maley F, Trimble RB, Tarentino AL, Plummer TH. (1989) Characterization of glycoproteins and their associated oligosaccharides through the use of endoglycosidases. Analytical Biochemistry 180:195–204. 2510544 10.1016/0003-2697(89)90115-2

[8] Tarentino AL, Maley F. (1976) Purification and properties of an endo-beta-N-acetylglucosaminidase from henoviduct. Journal of Biological Chemistry 251(21): 6537–6543. 977586

[9] Miyazono K, Thyberg J, Heldin CH. (1992) Retention of the transforming growth factor-β1 precursor in the Golgi complex in a latent endoglycosidase H-sensitive form. Journal of Biological Chemistry 267: 5668–5675. 1544940

[10] Tarentino AL, Maley F. (1974) Purification and properties of an endo-beta-N-acetylglucosaminidase from Streptomyces griseus. J Biol Chem 249(3):811–817. 4204552

[11] Mowla SJ, Farhadi HF, Pareek S, Atwal JK, Morris SJ, Seidah NG, et al (2001) Biosynthesis and post-translational processing of the precursor to brain-derived neurotrophic factor. Journal of Biological Chemistry 276: 12660–12666. doi: 10.1074/jbc.M008104200 11152678 10.1074/jbc.M008104200

[12] Frisch E, Kaup M, Egerer K, Weimann A, Tauber R, Berger M, et al (2011) Profiling of endo H released serum N-glycans using CE-LIF and MALDI-TOF-MS—application to rheumatoid arthritis. Electrophoresis 32: 3510–3515. doi: 10.1002/elps.201100250 22180205 10.1002/elps.201100250

[13] Freeze HH, Kranz C. (2010) Endoglycosidase and glycoamidase release of N-linked glycans. Curr Protoc Mol Biol. Chapter 17:Unit 17.13A. doi: 10.1002/0471142727.mb1713as89 20069534 10.1002/0471142727.mb1713as89 PMC3869378

[14] Wang F, Wang X, Yu X, Fu L, Liu Y, Ma L, et al (2015) High-level expression of endo-β-N-acetylglucosaminidase H from Streptomyces plicatus in *Pichia pastoris* and its application for the deglycosylation of glycoproteins. PLoS ONE 10(3): e0120458 doi: 10.1371/journal.pone.0120458 25781897 10.1371/journal.pone.0120458 PMC4362766

[15] Outchkourov NS, Roeffen W, Kaan A, Jansen J, Luty A, Schuiffel D, et al (2008) Correctly folded Pfs48 ⁄ 45 protein of Plasmodium falciparum elicits malaria transmission-blocking immunity inmice. Proc Natl Acad Sci USA 105: 4301–4305. doi: 10.1073/pnas.0800459105 18332422 10.1073/pnas.0800459105 PMC2393789

[16] Chen P, Wang C, Soong S, To K. (2003) Complete sequence of the binary vector pBI121 and its application in cloning T-DNA insertion from transgenic plants. Mol. Breed. 11, 287–293.

[17] Voinnet O, Rivas S, Mestre P, Baulcombe D. (2003) An enhanced transient expression system in plants based on suppression of gene silencing by the p19 protein of tomato bushy stunt virus. Plant J 33: 949– 956. 12609035 10.1046/j.1365-313x.2003.01676.x

[18] Milek RL, DeVries AA, Roeffen WF, Stunnenberg H, Rottier PJ, Konings RN, et al (1998) Plasmodium falciparum: heterologous synthesis of the transmission-blocking vaccine candidate Pfs48 ⁄ 45 in recombinant vaccinia virus-infected cells. Exp. Parasitol 90:165–174. doi: 10.1006/expr.1998.4315 9769246 10.1006/expr.1998.4315

[19] Sainsbury F, Thuenemann EC, Lomonossoff GP (2009) pEAQ: versatile expression vectors for easy and quick transient expression of heterologous proteins in plants. Plant Biotechnol 7(7):682–93. doi: 10.1111/j.1467-7652.2009.00434.x 19627561 10.1111/j.1467-7652.2009.00434.x

[20] Carter R, Bushell G, Saul A, Graves PM, Kidson C (1985) Two apparently nonrepeated epitopes on gametes of Plasmodium falciparum are targets of transmission-blocking antibodies. Infect Immun 50(1):102–6. 2412959 10.1128/iai.50.1.102-106.1985 PMC262142

[21] Klimyuk V, Pogue G, Herz S, Butler J, Haydon H (2014) Production of recombinant antigens and antibodies in Nicotiana benthamiana using 'magnifection' technology: GMP-compliant facilities for smalland large-scale manufacturing. Curr Top Microbiol Immunol 375: 127–154. doi: 10.1007/82_2012_212 22527176 10.1007/82_2012_212

[22] Křenek P, Šamajová O, Luptovčiak I, Doskočilová A, Komis G, Šamaj J (2015) Transient plant transformation mediated by Agrobacterium tumefaciens: Principles, methods and applications. Biotechnol Adv 33(6 Pt 2): 1024–1042. doi: 10.1016/j.biotechadv.2015.03.012 25819757 10.1016/j.biotechadv.2015.03.012

[23] Rybicki EP (2010) Plant-made vaccines for humans and animals. Plant Biotechnol J 8: 620–637. doi: 10.1111/j.1467-7652.2010.00507.x 20233333 10.1111/j.1467-7652.2010.00507.x PMC7167690

[24] Thuenemann EC, Lenzi P, Love AJ, Taliansky M, Bécares M, Zuñiga S, et al (2013) The use of transient expression systems for the rapid production of virus-like particles in plants. Curr Pharm Des 19:5564–5573. 23394559 10.2174/1381612811319310011

[25] Yusibov VM, Mamedov TG. (2010) Plants as an alternative system for expression of vaccine antigens. Proc. ANAS (Biol. Sci.) 65:195–200.

[26] Theisen M, Jore MM, Sauerwein R. (2017) Towards clinical development of a Pfs48/45-based transmission blocking malaria vaccine. Expert Rev Vaccines 9:1–8. doi: 10.1080/14760584.2017.1276833 PMID: 28043178 10.1080/14760584.2017.1276833 28043178

[27] Rener J et al (1983) Target Antigens of Transmission-Blocking Immunity on Gametes of *Plasmodium falciparum*. J. Exp. Med 158: 976–981. 6350527 10.1084/jem.158.3.976 PMC2187091

[28] Targett GAT, Harte PG, Eida S, Rogers NC, Ong CSL (1990) Plasmodium falciparum sexual stage antigens: immunogenicity and cellmediated responses. Immunol Let 25:77–82. doi: 10.1016/0165–2478(90)90095-8 1704351 10.1016/0165-2478(90)90095-8

[29] Vermeulen AN, Ponnudurai T, Beckers PJA, Verhave JP, Smits MA, Meuwissen JHE (1985) Sequential expression of antigens on sexual stages of Plasmodium falciparum accessible to transmissionblocking antibodies in the mosquito. J Exp Med 162:1460–1476. doi: 10.1084/jem.162.5.1460 2865324 10.1084/jem.162.5.1460 PMC2187939

[30] Carter R, Coulson A, Bhatti S, Taylor BJ, Elliott JF. (1995) Predicted disulfide-bonded structures for three uniquely related proteins of *Plasmodium falciparum*, Pfs230, Pfs48/45 and Pf12.Mol Biochem Parasitol 71(2): 203–210. 7477102 10.1016/0166-6851(94)00054-q

[31] Carter R, Graves PM, Keister DB, Quakyi IA (1990) Properties of epitopes of Pfs 48/45, a target of transmission blocking monoclonal antibodies, on gametes of different isolates of Plasmodium falciparum. Parasite Immunol 12:587–603. doi: 10.1111/j.1365-3024.1990.tb00990.x 1707506 10.1111/j.1365-3024.1990.tb00990.x

[32] Jones CS, Luong T, Hannon M, Tran M, Gregory JA, Shen Z, et al (2013) Heterologous expression of the C-terminal antigenic domain of the malaria vaccine candidate Pfs48/45 in the green algae *Chlamydomonas reinhardtii*. Appl Microbiol Biotechnol 97(5):1987–95. doi: 10.1007/s00253-012-4071-7 22592550 10.1007/s00253-012-4071-7

[33] Outchkourov N, Vermunt A, Jansen J, Kaan A, Roeffen W, Teelen K, et al (2007) Epitope analysis of the malaria surface antigen pfs48/45 identifies a subdomain that elicits transmission blocking antibodies. J Biol Chem 282: 17148–17156. doi: 10.1074/jbc.M700948200 17426022 10.1074/jbc.M700948200

[34] Rayon C, Lerouge P, Faye L (1998) The protein N-glycosylation inplants. J Exp Bot 49: 1463–1472.

